# Cultivating oyster mushrooms on red grape pomace waste enhances potential nutritional value of the spent substrate for ruminants

**DOI:** 10.1371/journal.pone.0246992

**Published:** 2021-02-16

**Authors:** Godfrey Mhlongo, Caven Mguvane Mnisi, Victor Mlambo

**Affiliations:** 1 Department of Animal Science, Faculty of Natural and Agricultural Science, North-West University, Mafikeng, South Africa; 2 Food Security and Safety Niche area, Faculty of Natural and Agricultural Science, North-West University, Mafikeng, South Africa; 3 School of Agricultural Sciences, Faculty of Agriculture and Natural Sciences, University of Mpumalanga, Nelspruit, South Africa; University of Illinois, UNITED STATES

## Abstract

The use of red grape pomace (GP; *Vitis vinifera* L. var. Shiraz) as a source of beneficial bioactive compounds in ruminant diets is limited by high levels of indigestible compounds in the grape skin matrix. This problem demands innovative, inexpensive, and easy-to-use strategies that improve the digestibility of GP. The bioconversion of GP using edible oyster mushrooms (*Pleurotus ostreatus*) is one such strategy that has not been previously explored. Therefore, this study evaluated the effect of cultivating oyster mushrooms on GP on chemical composition and *in vitro* ruminal fermentation parameters of the spent mushroom substrate. The GP was inoculated with oyster mushroom spawns at 0, 200, 300, 400, or 500 g/kg, and incubated for 4 weeks. Organic matter, acid detergent lignin, sodium, manganese, cobalt, and copper linearly declined (*P* < 0.05) as spawn rates increased. A quadratic trend was observed for crude protein, neutral detergent fibre, acid detergent fibre, magnesium, phosphorus, and calcium content in response to increasing spawn rates. Higher spawning rates (20–50%) had a positive effect (*P* < 0.05) on gas production from the immediately fermentable fraction (*a*), rate of gas production from the slowly fermentable fraction (*c*) and effective gas production. However, gas production from the slowly fermentable fraction (*b*) and potential gas production linearly declined in response to increasing spawning rates. There was a linear increase (*P* < 0.05) in the immediately degradable fraction (*a*), while quadratic effects were observed for partition factors, effective degradability, and *in vitro* organic matter degradability at 48 h in response to spawning rates. It can be concluded that inoculating GP with oyster mushroom spawn reduced fibre content while increasing crude protein content and *in vitro* ruminal fermentation efficiency of red grape pomace. Based on the quadratic responses of partition factors at 48 hours post-inoculation, the optimum spawning rate for maximum ruminal fermentation efficiency of GP was determined to be 300 g/kg.

## Introduction

To ensure that ruminant production complements other food production systems, it is important that they are reared on feed resources that have no direct food value for humans. This can be achieved by using non-conventional and low-cost feedstuffs such as red grape pomace (GP). In South Africa, the largest proportion of grapes is pressed to produce grape juice and wine, generating large quantities of GP that is discarded as a waste product [1]. The disposal of GP using traditional methods such as incineration and deposition on landfills has detrimental effects on the environment [2]. The disposal of GP into landfills might cause plant growth interference and water body contamination through the eutrophication process (the build-up of chemicals in water streams). Furthermore, Bustamante et al. [3] reported that red GP contains a large amount of biodegradable organic matter that might cause environmental hazards and pollution if not discarded properly. Consequently, it is important to find alternative uses of red GP that can improve its value while reducing the negative impact it has on the environment. Thus, it is important to identify alternative uses of GP to enhance environmental sustainability of the grape juice and wine-producing industries. Red grape pomace consists of a wide range of phenolic compounds, natural cellulose, minerals, polyunsaturated fatty acids, and phytochemicals that can be exploited to improve the performance and health status of ruminants [4]. Indeed, Teixeira et al. [5] observed that the compounds in GP have nutritional, anthelmintic, antioxidant, antimethanogenic and antimicrobial properties. However, Dwyer et al. [2] reported that about 3% of GP is reused as animal feed globally, possibly due to nutritional limitations imposed by antinutritional components such as condensed tannins and non-digestible fibre in GP. Condensed tannins are known to negatively affect nutrient digestibility because they inhibit cellulolytic and proteolytic enzymes as well as the growth of rumen bacteria [6]. The GP cell wall contains high amounts of insoluble proanthocyanidins, lignin, pectin substances and phenols that pose a problem for ruminant digestion processes. Thus, the use of GP as a ruminant feed is limited by high levels of lignified cell wall fraction and tannin content [7], which can adversely affect nutrient utilization when fed at high levels. Indeed, Baumgartel et al. [8] determined that OM and NDF digestibility of red grape pomace in sheep was rather low at 32 and 15%, respectively. The poor digestibility of GP was also corroborated by Guerra-Rivas et al. [9] *in sacco* using rumen cannulated sheep. Phenols and other potentially beneficial bioactive compounds in GP are covalently bound to the complex polysaccharides in the cell walls [10]. Consequently, poor digestibility of GP in ruminants reduces the bioavailability of bioactive compounds to the ruminant. Therefore, there is a need to identify and evaluate lignin degradation methods such as the use of fungi like white rot or mushrooms that can improve the digestibility of GP.

Solid-state fermentation with fungi is generally regarded as an economically and environmentally sustainable strategy for the bioconversion of lignin-rich substrates [11]. Indeed, Saratale et al. [12] reported that the use of white-rot fungi is cost-effective, requires low energy requirements and mild environmental conditions while efficiently degrading cellulose and lignin content of plant cell walls. Consequently, the use of fungi such as white rot and mushrooms to breakdown the fibre matrix has the potential to valorise GP as a ruminant feed ingredient. However, this approach has not been evaluated for GP. Enhancing the digestibility of GP can produce meat with human-health-promoting properties while mitigating environmental challenges that result from the disposal of GP and enteric methane emissions. In addition, edible mushrooms can also be a direct source of food thus their use in enhancing ruminant feeds is easier to sell to small scale farmers. No studies have investigated the use of oyster mushroom spawn to enhance the nutritive value of red GP for ruminants. Therefore, this preliminary study was designed to determine the effect of different oyster mushroom spawn rates on chemical composition and *in vitro* ruminal fermentation parameters of red grape pomace. We hypothesized that using red grape pomace as an oyster mushroom substrate will reduce the lignin content of the spent substrate and thus improve its ruminal fermentation compared to untreated substrates.

## Materials and methods

### Study area and resources

The study was carried out between July and August of 2019 at Molelwane Research Farm (25°86′00″S, 25°64′52″E) of the North-West University (North West province, South Africa). Fresh red grape pomace was acquired from Blaauwklipan Wine Estate and processed as described by Kumanda et al. [1]. The oyster mushroom spawn (*P*. *ostreatus*) was supplied by Eco-Agro Enterprise (PTY) LTD (Mbombela, South Africa).

### Substrate preparation and inoculation

The GP substrate was prepared according to the modified method of Shah et al. [13]. The GP substrate was sterilized in an autoclave (121°C for 1 hour at 100 kPa) and cooled at room temperature as described by Tuyen et al. [14]. Red grape pomace substrate (500 g per pot) was weighed into a total of 50 pots (223 cm^3^), which were designated as experimental units. Subsequently, the pots were randomly inoculated with the oyster mushroom spawn at 0, 200, 300, 400 or 500 g/kg of red GP. The pots were covered with black plastic bags (to conserve the moisture which is required by the mushroom spores to reproduce) and then kept at room temperature (20–25°C) and 70–80% relative humidity for the duration (4 weeks) of the experiment. Relative humidity was measured every other day using a multi-meter (HTC-1, Xuzhou Sanhe Automatic Control Equipment Co. Ltd, China). The inoculated GP substrates were sprinkled with 1.5 litres of water on an interval of 2 days to keep the substrate moist and humid. Red grape pomace substrate in pots that received no oyster mushroom spawn was similarly watered and incubated for 28 days.

### Experimental design and preparation of spent mushroom substrates (SMS)

The experiment was carried out in a completely randomized design in which each of the five spawning rates under investigation were replicated 10 times. About 125 g of the spent mushroom substrate (SMS) was sampled from each replicate pot at end of the incubation period (day 28). Mycelia were removed by hand and the SMS was oven-dried at 60°C until constant weight. The oven-dried SMS was milled to pass through a 1 mm sieve (Polymix PX-MFC 90 D, Switzerland) and stored in labelled sample bottles pending chemical analysis and *in vitro* ruminal fermentation.

### Chemical analyses

The Official Analytical Chemists International methods [15] were used to analyse the SMS for dry matter (method no. 930.15), organic matter (method no. 942.05) and crude protein (method no. 984.13). Neutral detergent fibre (NDF) and acid detergent fibre (ADF) were determined by refluxing milled SMS samples (0.45–0.5 g) with the neutral detergent and acid detergent solutions for 60 min and 75 min, respectively, using the ANKOM 2000 fibre analyser (ANKOM Technology, New York) according to the method described by Van Soest et al. [16]. The NDF was determined using a heat-stable α-amylase but without sodium sulphite. Acid detergent lignin (ADL) content was determined by difference after treating ADF residues in ANKOM F57 bags with 72% sulfuric acid to dissolve cellulose. The fibre components were expressed in g/kg DM with the inclusive of residual ash. Mineral content (Na, Mg, P, S, Ca, Mn, Co, Cu and Zn) was determined following the guidelines provided by Agri-Laboratory Association of Southern Africa [17].

### *In vitro* ruminal gas production

The *in vitro* ruminal gas production parameters were determined using the Reading Pressure Technique (RPT) developed by Mauricio et al. [18]. Rumen inoculum was collected in the morning from a rumen fistulated Bonsmara donor cow (~600 kg live-weight) that was cared for according to procedures approved (NWU-00126-13-A9) by the Animal Production Research Ethics Committee of the North-West University, South Africa. The rumen inoculum was collected and processed as described by Mnisi & Mlambo [19]. Milled SMS were weighed (1 ± 0.001 g) and placed into 125 ml glass serum bottles to which 90 ml of the ANKOM buffer solution was added to each of the bottles. The serum bottles were purged with CO_2_ gas before being sealed with rubber stoppers and transferred into an incubator set at 39°C overnight. In the morning, serum bottle contents were each inoculated with 10 ml of rumen fluid through the rubber stoppers using a syringe and incubated for 48 h. Headspace gas pressure was measured using a pressure transducer (model PX4200-015GI, Omega Engineering, Inc., Laval, QC, Canada) at 12, 24, 36 and 48 h after inoculation. The gas pressure readings (psi) were then converted to the gas volume (ml) using the following site-specific equation: *y* = 0.034*x*^2^+6.2325*x*+1.8143, where *y* = gas volume (ml) and *x* = measured gas pressure (psi). Cumulative gas production data were fitted into the Ørskov & McDonald [20] non-linear model: *Y* = *a* + *b*(1−*e*^−*c*(*t*)^), to determine *a* (gas production from the immediately fermentable fraction, *b* (the gas production from the slowly fermentable fraction), *c* (the gas production rate constant for the insoluble fraction, *b*). Potential gas production (*PGas*) was calculated as the summation of fractions *a* and *b*, whereas the effective gas production (*EGas)* was calculated using the following formula: *EGas* = *a* + $+\frac{bc}{k+c}$, where *k* is the rumen outflow rate assumed to be 2% per hour.

### *In vitro* ruminal dry matter degradability

The *in vitro* ruminal dry matter degradability (DMD) of SMS was determined using the Daisy^II^ incubator according to the ANKOM method for the *in vitro* true digestibility (ANKOM Technology, 2001). The rumen fluid used for this step was collected and processed as already described for the *in vitro* ruminal gas production investigation above. Exactly 400 ml of the processed rumen fluid was used to inoculate each of the four Daisy^II^ incubator jars containing the ANKOM F57 filter bags with SMS samples (0.45–0.5 g) and 1600 ml pre-warmed buffer solution (39°C). The jars were continuously purged with CO_2_ to maintain anaerobic conditions before they were closed and placed in the incubation chamber (39°C). The ANKOM F57 filter bags were incubated for a maximum period of 72 h, punctuated by periodic (2, 4, 8, 12, 24, 36, 48 and 72 h) withdrawals of bags to determine dry matter degradability. The withdrawn bags were washed with cold water for 20 minutes before being dried at a temperature of 105°C for 12 h. The 0-h samples were washed and oven-dried in the same manner as the incubated samples. The DMD data was fitted into the Ørskov & McDonald [20] non-linear model using the: *Y* = *a* + *b*(1−*e*^−*c*(*t*)^), to determine *a* (the immediately degradable fraction), *b* (the slowly degradable fraction), *c* (rate of degradation of the slowly degradable fraction, *b*). Potential degradability (*PDeg*) was calculated as the summation of fractions *a* and *b*, whereas the effective degradability (*EDeg)* was calculated using the following formula: *EDeg* = $a+\frac{bc}{k+c}$, where *k* is the rumen outflow rate assumed to be 2% per hour. Potential OM degradability (*PDeg*) and effective OM degradability (*EDeg)* were calculated as described for *PGas* and *EGas* above. The *in vitro* ruminal organic matter degradability (*iv*OMD) was determined through the incineration of the ash-free ANKOM F57 filter bags with residues placed on pre-weighed crucibles using a muffle furnace set at 600°C for 12 h. Partition factors (ml/g OM), a measure of fermentation efficiency, were calculated as a ratio of the cumulative gas production to *iv*OMD at 12, 24, 36 and 48 h post-incubation.

### Data analysis

Response surface regression analysis (Proc RSREG; SAS [21]) was employed to describe linear and quadratic trends of GP’s chemical components and *in vitro* ruminal fermentation parameters in response to spawning rates as well as to determine the optimum spawning rate of the oyster mushroom. A one-way ANOVA (PROC GLM; SAS [21]) was used to analyse the chemical composition, *in vitro* ruminal fermentation data in a completely randomised design, where oyster mushroom spawn rate was the only factor. For all the statistical tests, significance was declared at *P* < 0.05. The least-squares means were compared using the probability of difference option in SAS.

## Results

### Chemical composition

Table 1 shows the chemical composition of red grape pomace spent oyster mushroom substrate. There were significant linear effects for ADL [*y* = 574.0 (±22.59) - 4.63 (±1.78)*x*; R^2^ = 0.20; *P* = 0.019] while quadratic trends were observed for NDF [*y* = 0.06 (±0.012)*x*² - 3.9 (±0.65)*x* + 688.1 (±8.25); R^2^ = 0.53; *P* = 0.0001], ADF [*y* = 0.05 (±0.011)*x*² - 3.9 (±0.590)*x* + 627.0 (±7.49); R^2^ = 0.65; *P* = 0.0001] and CP [*y* = 108.1 (±2.21) + 1.04 (±0.191)*x*—0.01 (±0.004)*x*^2^; R^2^ = 0.52; *P* = 0.0004] in response to the increasing oyster mushroom spawning rates. For minerals, there were negative linear trends (*P* < 0.05) for sodium [R^2^ = 0.43; *P* = 0.018], manganese [R^2^ = 0.49; *P* = 0.008], cobalt [R^2^ = 0.46; *P* = 0.018] and copper [R^2^ = 0.55; *P* = 0.004] in response to spawning rates of oyster mushroom. However, positive quadratic trends were observed for magnesium [R^2^ = 0.45; *P* = 0.014], phosphorus [R^2^ = 0.38; *P* = 0.027] and calcium [R^2^ = 0.42; *P* = 0.037] content in response to increasing spawning rates of oyster mushroom. There were no DM differences across mushroom spawning rate treatments. Uninoculated pomace (GP0) had a similar OM concentration as substrates GP20, GP30 GP40 and GP50. Substrate GP0 had a higher ADF content (632.9 g/kg DM) than substrates GP20 (551.1 g/kg DM), GP30 (559.9 g/kg DM), GP40 (564.5 g/kg DM) and GP50 (552.0 g/kg DM). Substrate GP0 had a higher ADL content (595.0 g/kg DM) than substrate GP30 (487.7 g/kg DM), GP40 (501.8 g/kg DM) and GP50 (508.8 g/kg DM). Substrate GP20 had a higher CP content (126.9 g/kg DM) compared to substrates GP0 (107.3 g/kg DM).

**Table 1:** Red grape pomace spent mushroom substrate chemical composition (g/kg DM, unless stated otherwise) in response to graded levels of oyster mushroom spawn.

	^1^Substrates						*P* value	
Parameters	GP0	GP20	GP30	GP40	GP50	^2^SEM	Linear	Quadratic
Dry matter (g/kg)	936.5	937.2	941.2	938.1	938.1	1.23	0.283	0.121
Organic matter	859.7^ab^	857.7^a^	863.5^ab^	861.6 ^ab^	864.4^b^	1.50	0.006	0.328
Neutral detergent fibre	692.0^c^	615.4^a^	629.5^ab^	638.5^b^	633.7^ab^	5.74	0.000	0.000
Acid detergent fibre	632.9^b^	551.1^a^	559.9^a^	564.5^a^	552.0^a^	5.15	0.000	0.000
Acid detergent lignin	595.0^b^	522.2^ab^	487.7^a^	501.8^a^	508.8^a^	21.13	0.019	0.059
Crude protein	107.3^a^	126.9^b^	124.1^b^	125.4^b^	126.4^b^	2.16	0.000	0.000
Sodium	4.24^bc^	4.38^c^	3.12^ab^	3.79^abc^	2.98^a^	0.29	0.018	0.388
Magnesium	2.31^bc^	2.81^c^	1.99^b^	2.32^bc^	1.72^a^	0.20	0.644	0.014
Phosphorus	1.83^ab^	2.21^b^	1.54^a^	1.83^ab^	1.54^a^	0.11	0.610	0.027
Sulphur	1.98	2.49	1.93	2.05	1.50	1.25	0.788	0.487
Calcium	30.9^a^	39.7^b^	30.2^a^	35.7^ab^	31.3^a^	2.01	0.161	0.037
Manganese (mg/kg DM)	30.0^b^	20.0^a^	20.0^a^	19.0^a^	19.0^a^	0.001	0.008	0.946
Cobalt (mg/kg DM)	0.40	0.20	0.20	0.10	0.20	0.000	0.018	0.227
Copper (mg/kg DM)	13.0^c^	12.0^bc^	8.0^ab^	8.0^ab^	7.0^a^	0.001	0.004	0.872
Zinc (mg/kg DM)	30.0	20.0	20.0	20.0	20.0	0.002	0.261	0.759

^a,b,c^ In a row, different superscripts denote significant differences (*P* < 0.05) between spawning rate means.

^1^Substrates: GP0 = uninoculated red grape pomace; GP20 = red grape pomace inoculated with oyster mushroom spawn at 200 g/kg; red grape pomace inoculated with oyster mushroom spawn at 300 g/kg; GP40 = red grape pomace inoculated oyster mushroom spawn at 400 g/kg; GP50 = red grape pomace inoculated with oyster mushroom spawn at 500 g/kg.

^2^SEM = standard error of the mean.

### *In vitro* ruminal gas production

Table 2 shows that increasing spawning rates resulted in linear increases in fractions *a* [*y* = 0.04 (±0.037)*x* + 4.4 (±0.44); R^2^ = 0.40; *P* = 0.000], *c* [*y* = 0.0007 (±0.00023)*x* + 0.02 (±0.003); R^2^ = 0.21; *P* = 0.018], and *EGas* [*y* = 0.13 (±0.05)*x* + 12.0 (±0.54); R^2^ = 0.21; *P* = 0.009], but linearly decreased fractions *b* [*y* = 18.1 (±1.05) - 0.12 (±0.089)*x*; R^2^ = 0.32; *P* = 0.000] and *PGas* [*y* = 22.5 (±1.12) - 0.09 (±0.095)*x*; R^2^ = 0.094; *P* = 0.037]. Positive quadratic trends (*P* < 0.05) were observed for fractions *c* and *EGas* in response to the increasing oyster mushroom spawning rates.

**Table 2:** *In vitro* ruminal gas production parameters of red grape pomace spent oyster mushroom substrate.

	^1^Substrates						Significance	
^2^Parameters	GP0	GP20	GP30	GP40	GP50	^3^SEM	Linear	Quadratic
*a*	4.27^a^	6.02^b^	5.44^ab^	6.43^bc^	7.80^c^	0.425	0.000	0.468
*b*	18.11^b^	15.56^ab^	14.54^ab^	13.18^a^	12.26^a^	1.035	0.000	0.922
*c* (%/h)	0.016^a^	0.024^ab^	0.029^b^	0.028^ab^	0.026^ab^	0.003	0.018	0.036
*PGas*	22.38	21.58	19.98	19.62	19.03	1.103	0.037	0.848
*EGas*	11.85^a^	14.43^b^	13.91^ab^	13.71^ab^	13.64^ab^	0.562	0.009	0.477

^a,b,c^ In a row, different superscripts denote significant differences (*P* < 0.05) between spawning rates.

^1^Substrates: GP0 = uninoculated red grape pomace; GP20 = red grape pomace inoculated with oyster mushroom spawn at 200 g/kg; red grape pomace inoculated with oyster mushroom spawn at 300 g/kg; GP40 = red grape pomace inoculated oyster mushroom spawn at 400 g/kg; GP50 = red grape pomace inoculated with oyster mushroom spawn at 500 g/kg.

^2^Parameters: *a* = the immediate fermentation fraction; *b* = the slowly fermentable fraction; *c* = fermentation rate of fraction (*b*); *PGas* = potential gas production; *EGas* = effective gas production.

^3^SEM = standard error of the mean.

### *In vitro* ruminal dry matter degradability

Table 3 shows that the immediately degradable DM fraction *a* [*y* = 3.6 (±1.69)*x* + 13.9 (±23.13); R^2^ = 0.59; *P* = 0.042] linearly increased while *EDeg* [*y* = 3.2 (±1.05)*x*—0.05 (±0.018)*x*² + 87.4 (±14.42); R^2^ = 0.65; *P* = 0.048] responded quadratically to increasing spawning levels. Neither linear nor quadratic trends (*P* > 0.05) were observed for the slowly degradable DM fraction (*b*), rate constant (*c*), as well as *PDeg* as oyster mushroom spawn levels increased.

**Table 3:** *In vitro* ruminal dry matter degradability (g/kg DM) of red grape pomace spent oyster mushroom substrate.

	^1^Substrates						*P*-value	
^2^Parameters	GP0	GP20	GP30	GP40	GP50	^3^SEM	Linear	Quadratic
*a*	23.98^a^	49.89^ab^	90.58^bc^	92.47^bc^	101.32^c^	0.587	0.042	0.214
*b*	71.19^a^	85.76^a^	87.49^a^	277.8^b^	71.06^a^	0.129	0.422	0.717
*c* (%/h)	0.980^b^	0.536^b^	0.029^b^	0.004^a^	0.022^b^	0.413	0.093	0.650
*PDeg*	86.01^a^	135.65^a^	178.1^a^	364.7^b^	172.4^a^	0.514	0.065	0.301
*EDeg*	92.38^a^	125.58^b^	147.5^b^	133.5^b^	138.8^b^	0.648	0.068	0.048

^a,b,c^ In a row, different superscripts denote significant differences (*P* < 0.05) between spawning rates.

^1^Substrates: GP0 = uninoculated red grape pomace; GP20 = red grape pomace inoculated with oyster mushroom spawn at 200 g/kg; red grape pomace inoculated with oyster mushroom spawn at 300 g/kg; GP40 = red grape pomace inoculated oyster mushroom spawn at 400 g/kg; GP50 = red grape pomace inoculated with oyster mushroom spawn at 500 g/kg.

^2^Parameters: *a* = the immediately degradable fraction; *b* = the slowly degradable fraction; *c* = degradation rate of fraction *b*; *PDeg* = potential degradability; *EDeg* = effective degradability.

^3^SEM = standard error of the mean.

Table 4 shows quadratic trends for OMD36 [*y* = 0.095 (±0.036)*x*² - 6.800 (±2.070)*x* + 878.54 (±28.388); R^2^ = 0.69; *P* = 0.040], OMD48 [*y* = 0.046 (±0.011)*x*² +2.800 (±0.621)*x* + 771.9 (±8.522); R^2^ = 0.76; *P* = 0.006], and PF48 [*y* = 0.00009 (±0.00002)*x*² +0.005 (±0.000097)*x* + 0.171 (±0.013); R^2^ = 0.77; *P* = 0.006] in response to incremental levels of the oyster mushroom spawn. There were no significant differences in terms of OMD12, OMD24 and OMD48 of the substrates. Uninoculated substrate (GP0) had lower PF48 compared to the oyster mushroom spawn inoculated substrates, whose own PF values did not differ (*P* > 0.05).

**Table 4:** Organic matter degradability (g/kg OM) and partition factors (ml/mg OM) of red grape pomace spent oyster mushroom substrate harvested at four weeks post-inoculation.

	^1^Substrates						*P* value	
^2^Parameters	GP0	GP20	GP30	GP40	GP50	^3^SEM	Linear	Quadratic
OMD12	804.9	766.5	775.0	764.9	773.9	0.149	0.531	0.466
OMD24	804.5	751.1	761.6	772.3	515.7	0.377	0.194	0.268
OMD36	859.8^b^	777.4^a^	772.7^a^	793.2^a^	763.4^a^	0.689	0.044	0.040
OMD48	775.1	773.8	729.4	745.3	727.2	0.773	0.133	0.006
PF12	0.094^a^	0.167^b^	0.163^ab^	0.123^ab^	0.156^ab^	0.261	0.573	0.232
PF24	0.100^a^	0.208^b^	0.189^ab^	0.162^ab^	0.170^ab^	0.383	0.505	0.123
PF36	0.059^a^	0.136^ab^	0.168^b^	0.111^ab^	0.169^b^	0.278	0.450	0.245
PF48	0.161^a^	0.215^b^	0.242^b^	0.221^b^	0.218^b^	0.824	0.452	0.002

^a,b^ In a row, different superscripts denote significant differences (*P* < 0.05) between spawning rates.

^1^Substrates: GP0 = uninoculated red grape pomace; GP20 = red grape pomace inoculated with oyster mushroom spawn at 200 g/kg; red grape pomace inoculated with oyster mushroom spawn at 300 g/kg; GP40 = red grape pomace inoculated oyster mushroom spawn at 400 g/kg; GP50 = red grape pomace inoculated with oyster mushroom spawn at 500 g/kg.

^2^Parameters: OMD12 = *in vitro* organic matter degradability at 12 h after inoculation; OMD24 = *in vitro* organic matter degradability at 24 h after inoculation; OMD36 = *in vitro* organic matter degradability at 36 h after inoculation; OMD48 = *in vitro* organic matter degradability at 48 h after inoculation; PF12 = partition factors at 12 h post-incubation; PF24 = partition factors at 24 h post-incubation; PF36 = partition factors at 36 h post-incubation; PF48 = partition factors at 48 h post-incubation.

^3^SEM = standard error of the mean.

## Discussion

### Chemical composition

The use of fungal treatments has been reported to be effective in reducing fibre constituents of substrates [22, 23]. Indeed, inoculation with oyster mushroom spawn reduced fibre content (NDF, ADF and ADL) of GP in the current study as expected. Increasing spawning rate linearly decreased the DM and OM content of GP substrates in this study. This suggests that when cultivated on GP, *P*. *ostreatus* is a non-selective fungus that breaks down both lignin and polysaccharides resulting in the loss of total dry matter of substrate. This was also in line with the results reported by Nasehi et al. [24] in pea straw subjected to solid-state fermentation after inoculation with *Pleurotus florida*. The loss of DM and OM from the GP substrate is inevitable because the mushrooms use it up as a nutrient source. This loss would be magnified where the mushrooms are harvested for human consumption while the spent pomace is used as an animal feed ingredient. However, where the mushrooms remain part of the spent substrate used for animal feeding, this loss will be marginal. Nevertheless, the DM and OM losses do not diminish the utility of this ingenious valorisation strategy because GP substrate is a waste product that is produced in very large quantities and disposed in ways that pose an environmental challenge hence the small biomass losses are an acceptable trade-off.

An increase in CP content with an increase in spawning level was observed in the present study. Previous studies [25, 26] also reported an increase in protein when agricultural by-products were treated with different strains of white-rot fungi. The increase in CP content of inoculated substrate can be explained by the hydrolysis of carbohydrates and their subsequent use as a carbon source to synthesize fungal biomass that is rich in protein [27]. Furthermore, Sallam et al. [28] reported that the increase in CP content might be due to mycelia enzymes that are secreted into the substrate during the fermentation process. In this study, inoculation with oyster mushroom spawn significantly reduced the high amount of lignin content in the GP, which hinders its effective utilization by rumen microbes. Indeed, GP fibre content (NDF, ADF and ADL) was lower in inoculated substrates relative to the control GP substrate, which agreed with the findings of Azim et al. [29] and Akinfemi et al. [30]. Yilka [31] suggested that the reduced NDF, ADF and ADL contents on the residues might be caused fungi’s ability to solubilise the plant cell walls and use them as carbon sources thereby changing the ratio of insoluble to soluble carbohydrates.

A slight decrease in macro-mineral content (except for Ca) was observed when higher levels of the oyster mushroom spawn were used to inoculate GP substrates. Sales-Campos et al. [32] also reported a slight decrease in the P and K content in sawdust, crushed sugarcane bagasse, *Ochroma piramidale* and crushed peach palm substrates when treated with *P*. *ostreatus*. This reduction in mineral content of spent substrate could be attributed to the action of basidiomata selectively removing nutrients for the process of fruiting body formation. However, incremental spawning rates showed positive quadratic effects on magnesium, phosphorus, and calcium content. These changes in macro-mineral content of SMS were minor and both inoculated and uninoculated substrates were within the required dietary mineral range for ruminants as recommended by NRC [33]. Except for selenium, micro-mineral content (iron, manganese, zinc, cobalt, and copper) declined in response to incremental levels of oyster mushroom spawns. These findings corroborate those reported by Lee et al. [34] for Mn, Co, Cu, Zn, and Se. The micro-minerals were below the required range for ruminants as reported by NRC [33], except for Cu for small ruminants and Fe for both small and large ruminants. This implies that ruminants are likely to suffer from micro-mineral deficiencies when fed spent GP substrates, thus trace minerals supplementation either in a form of lick blocks or as feed additives would be necessary.

### *In vitro* ruminal gas production

Gas production from the slowly degradable fraction (*b*) of spent GP quadratically responded to increasing spawning rates of oyster mushroom. Akinfemi [35] also reported a higher *b* fraction for the fungal-treated substrates compared to untreated ones, which might be associated with the reduction in lignin content making it possible for the rumen microbes to readily ferment cellulose and hemicellulose in treated substrates. Cone et al. [36] reported that in the initial stages of ruminal fermentation, the soluble components have the highest contribution towards total gas production while the insoluble but degradable components mainly contribute to the gas production of the second phase of fermentation. Oyster mushroom-inoculated substrates had a higher fermentation rate (*c*) than the uninoculated substrates because the lignolytic activity of oyster mushroom spawn increases the accessibility of cellulose and hemicellulose to rumen microbes [37, 38] resulting in a higher rate of fermentation. The use of oyster mushroom spawn to breakdown lignin in GP is an innovative strategy to valorise this waste by-product. Indeed, increasing spawning rate improved total gas production and rate of fermentation of GP substrate. However, while the higher amount and rate gas production indicate increased rumen microbial activity as more cellulose and hemicellulose became readily available for fermentation, these parameters on their own are not valid indicators of improved nutritive value. This is because fermentation gases such as methane and carbon dioxide are waste products that represent a loss of dietary energy thus higher gas production would be undesirable unless it is accompanied by higher levels of dry matter degradability. As a result, an index named partition factor (PF), which is calculated as a ratio of cumulative gas production to dry matter degradability in a given incubation period, was used in this study to evaluate the effect of fungal inoculation on *in vitro* ruminal fermentation efficiency of GP.

### *In vitro* ruminal dry matter degradability

Over the years, physical and chemical treatments have been shown to improve the degradability and intake of certain fibrous agricultural by-products in ruminants. However, concerns regarding safety, cost, and environmental impact of these traditional approaches tend to limit their application [39]. This has led to increased interest in the use of white-rot fungi to improve the nutritive value and *in vitro* dry matter degradability of certain agricultural by-products [40]. Results from this study show that increasing the spawning rate of the fungus caused a linear improvement in the immediately degradable fraction (*a*) and a quadratic effect on effective degradability (*EDeg*) as well as organic matter degradability at 36 hours of incubation (*iv*OMD36). These responses can be explained by the reduction in the concentration of cell wall components (NDF, ADF and ADL) upon oyster mushroom treatment of GP substrates. In particular, the reduction in lignin content allows rumen microbes easy access to GP components while reducing the inhibitory effect of lignin on microbial activity. This is supported by a study by Khattab et al. [41], where fungal treatment of rice straws reduced the fibre content thereby increasing nutrient bioavailability for rumen microbes.

Higher *a*, *b*, *PDeg* and *EDeg* fractions were observed in inoculated GP substrates than the control. This suggests that the reduction in lignin content of GP by the oyster mushroom reported above resulted in improved degradation of the spent substrate by the rumen microbes. The current results are supported by Valmaseda et al. [42] and Gutierrez et al. [43] who both noted a reduction in cell wall contents and increased soluble fractions of carbohydrates when wheat straws were fermented with *Pleurotus* fungi. For the same reasons, increasing spawn rates had a quadratic response on OMD36, OMD48 and fermentation efficiency (PF48). These results suggest that the oyster mushroom spawn was able to degrade structural carbohydrates resulting in efficient ruminal fermentation as observed by Colombatto et al. [44]. Indeed, Mlambo et al. [45] confirm that higher PF values indicate higher fermentation efficiency in the rumen. Thus, higher PF values at 36 and 48 h post-incubation of the treated substrates suggest that the fermentation of inoculated substrates resulted in more organic matter being degraded per unit change in gas produced. Based on the quadratic responses of partition factors at 48 hours post-inoculation, the optimum spawning rate for maximum ruminal fermentation efficiency of spent GP was determined to be 300 g/kg. It is envisaged that the spent GP substrate, together with the mushroom, could be incorporated into finishing diets of ruminants as a source of fibre, protein, and bioactive compounds. The spent GP substrate can be used directly after fungal fermentation or can be sun-dried before being added as part of total mixed rations for finishing animals. The amount of this valorised product that could be included in diets of ruminants will depend on factors such as type of animal, desired weight gain, other dietary ingredients, and age of animal. The chemical composition of the spent GP shows that it may not be used as a sole diet for any ruminant but as a fortified fibre source in combination with other feed ingredients.

## Conclusion

Inoculating red grape pomace with *P*. *ostreatus* spawn improved the crude protein and reduced the fibre content of the spent substrates. The results show that the oyster mushroom degraded lignin in red grape pomace and improved fermentation efficiency of the spent substrate. *In vitro* ruminal fermentation efficiency of red grape pomace was maximized when inoculated with mushroom spawn at a rate of 300 g/kg. It was concluded that cultivating *P*. *ostreatus* on red grape pomace is an effective strategy to improve the potential nutraceutical value of red grape pomace waste for ruminants.
